# Extramedullary Waldenström Macroglobulinemia Presenting as a Subcutaneous Penile Mass

**DOI:** 10.7759/cureus.17809

**Published:** 2021-09-07

**Authors:** Cassandra J Palmer, Joseph Sahagun, Alan G David, Cameron W Taylor, Yousef Al-Shraideh

**Affiliations:** 1 Department of Urology, University of Illinois College of Medicine-Peoria, Peoria, USA; 2 Department of Surgery, University of Illinois College of Medicine-Peoria, Peoria, USA; 3 Department of Cardiology, University of Illinois College of Medicine-Peoria, Peoria, USA; 4 Department of Diagnostic Radiology, Central Illinois Radiological Associates, University of Illinois College of Medicine-Peoria, Order of Saint Francis, Peoria, USA; 5 Department of Urology, University of Illinois College of Medicine-Peoria, Order of Saint Francis, Peoria, USA

**Keywords:** recurrence, waldenström macroglobulinemia, primary penile malignancy, uro-oncology, penile mass

## Abstract

Primary penile malignancy is a rare occurrence in the United States, with squamous carcinoma being the most common aetiology. Non-squamous penile cancers are scarcely reported in the literature. We present a unique case of a 65-year-old male with a history of Waldenström macroglobulinemia (WM) previously in remission complaining of a painless subcutaneous bump on the base of the penis. Biopsy with histological and immunohistochemical analysis confirmed the recurrence of WM. This novel case illustrates an unusual presentation of the disease after being successfully managed with chemotherapy and immunotherapy in an asymptomatic individual. There is only another reported case in the literature of a patient with a similar presentation. We highlight the clinical features and presentation of this condition, including a consensus for the approach and management of non-Hodgkin’s lymphomas of the penis.

## Introduction

Penile malignancy is rare and accounts for less than 1% of male cancers within the United States [1]. Nonetheless, penile cancers should be ruled out when encountering a penile lesion or mass to prevent sequelae from delayed treatment or potential escalation that would result in permanent cosmetic and functional deficits [2]. The most common type of penile malignancy is squamous cell carcinoma related to human papillomavirus, while metastases, soft tissue sarcomas, and lymphomas are less common causes [1]. Waldenström macroglobulinemia (WM) of the penis is rare, with only one other case reported in the literature that encompassed cutaneous and systemic manifestations, typical findings in delayed-onset WM [3]. Here, we report a case of WM relapse that was discovered in an asymptomatic individual after a histopathological workup of an insidious subcutaneous penile mass. This case represents a unique manifestation of extramedullary involvement in WM.

## Case presentation

**Figure 1:**
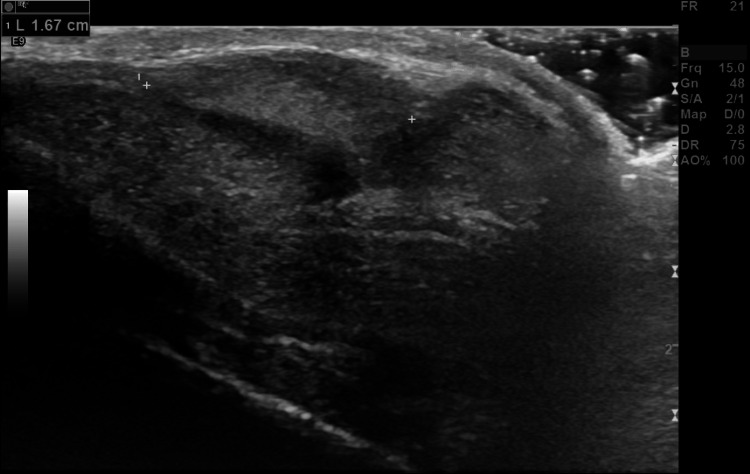
Ultrasound demonstrating a 1.67 cm slightly hyperechoic soft tissue mass overlying the medial aspect of the right corpus cavernosum. The mass is adjacent to the cavernosal tissue and measures 1.6 × 0.8 × 1.4 cm.

**Figure 2:**
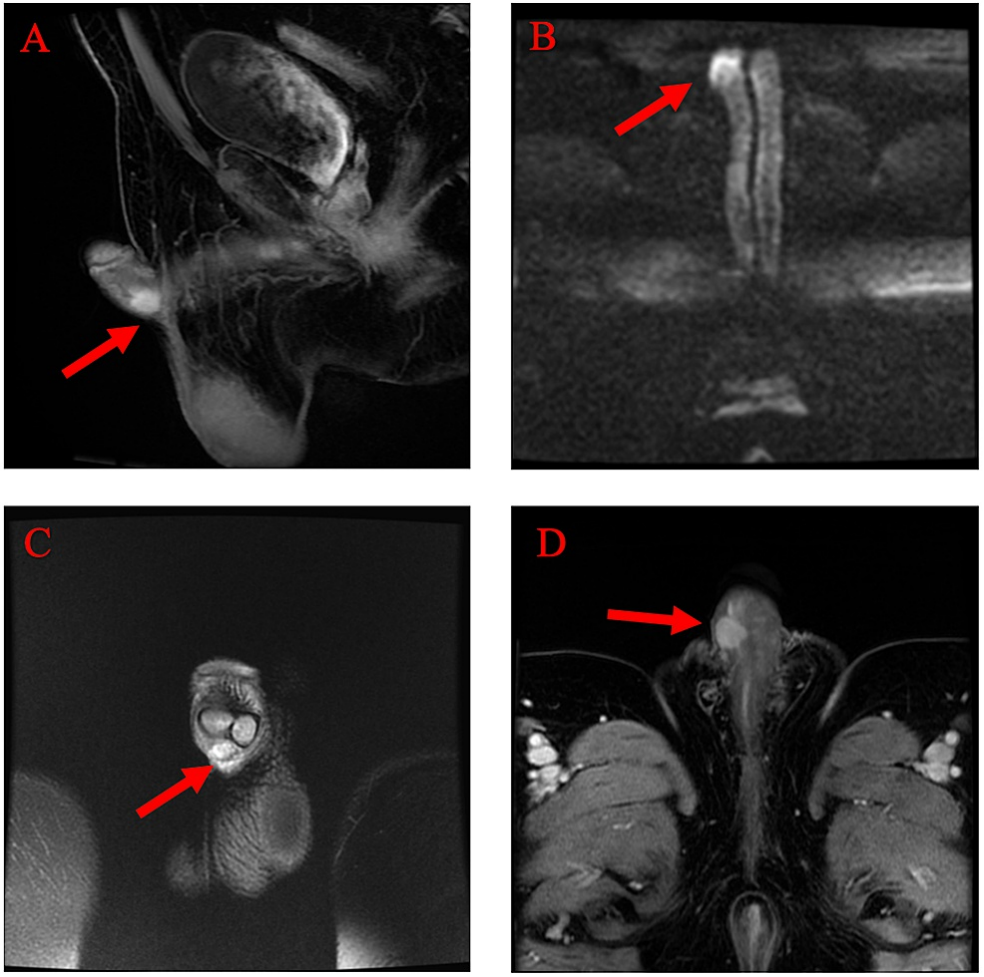
Radiological MRI imaging. (A) T2 fat saturation sagittal image demonstrating a well-circumscribed, T2 hyperintense mass at the ventral right aspect of the base of the penis. No definite involvement of the corpora cavernosum or spongiosum. (B) Diffusion-weighted images demonstrating focal diffusion restriction within the mass. (C and D) T1 postcontrast coronal and axial images demonstrating homogenously enhancing mass. MRI: magnetic resonance imaging

A 65-year-old male with a medical history significant for scleroderma, Raynaud’s phenomenon, benign prostatic hyperplasia status post-transurethral resection of the prostate, low-grade appendiceal mucinous neoplasm, and WM diagnosed six years ago and in remission since 2017 presented to outpatient urology with a right painless mass at the base of the penis that had been slowly increasing in size over six months. He was previously treated for WM with bendamustine that was completed in 2015, along with maintenance rituximab therapy until 2017. An ultrasound was performed that showed a 1.6 cm soft tissue nodule overlying the medial aspect of the right corpus cavernosum, near the base of the penis (Figure 1). A follow-up MRI of the pelvis with and without contrast demonstrated an enhancing lesion without invasion of the corpus cavernosum or corpus spongiosum (Figure 2). The lesion was excised with wide margins. Histopathology exhibited fibrous tissue with dense infiltration of small lymphocytes with scant cytoplasm, round nuclei, and condense nuclear chromatin that was CD20, CD23, and B-cell lymphoma 2 positive. The overall findings were indicative of low-grade B-cell lymphoma, consistent with a lymphoplasmacytic lymphoma (LPL). The treatment plan included the resumption of rituximab and ibrutinib until progression; this plan was chosen due to the favorable toxicity profile.

## Discussion

WM, a subtype of LPL, is a rare indolent (slow-growing) B-cell lymphoma that occurs in less than 2% of patients with non-Hodgkin lymphoma with an incidence rate of three per million [4]. The most common presenting symptom of WM is fatigue related to anaemia; other possible manifestations include hepatomegaly, splenomegaly, lymphadenopathy, or any sequelae related to hyperviscosity syndrome. WM typically affects hematologic tissues, including bone marrow, lymph nodes, and spleen. WM even more rarely affects extramedullary tissues, as seen in 4.4% of patients, with pulmonary involvement being the most common (30%). Other less common locations include soft tissue, cerebrospinal fluid, renal, and bone [5]. Due to its rare nature, the extramedullary involvement of WM is not well understood. This case is fascinating because of the unusual presentation as a penile mass.

**Figure 3:**
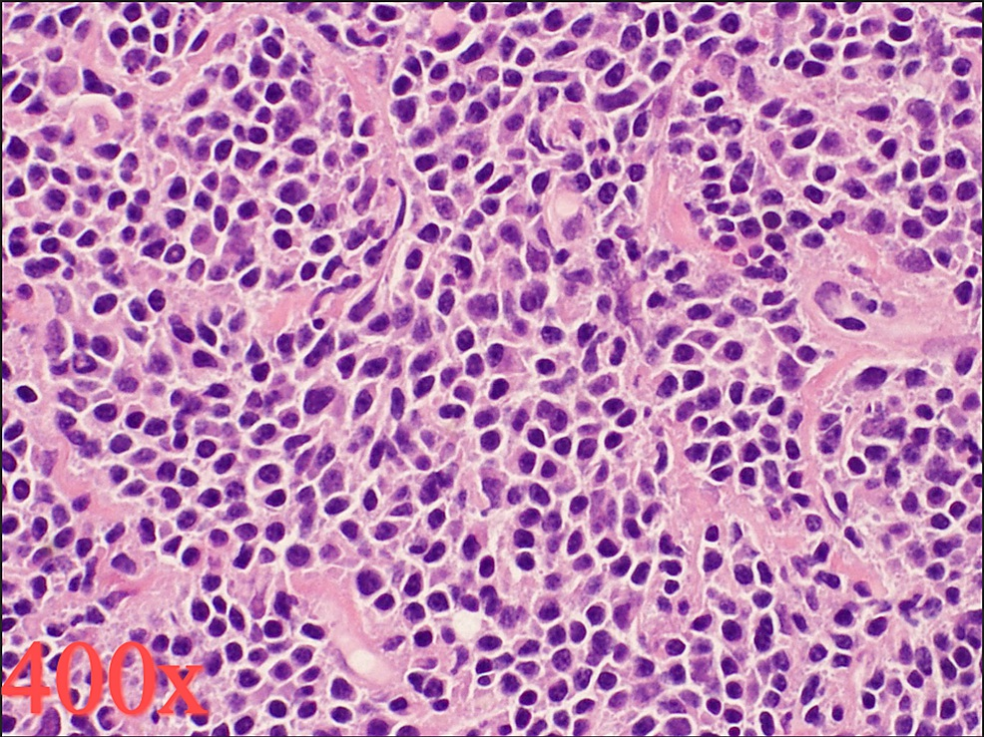
Histopathological findings. At lesion excision in April 2021, staining with hematoxylin and eosin revealed diffuse soft tissue infiltration by small B lymphocytes, plasmacytoid lymphocytes, and plasma cells.

All cutaneous presentations of WM are characterised in a 2001 report, describing the lesions as infiltrative papules and plaques with red-brown discolouration [6]. Cutaneous WM lesions can be characterised as neoplastic and non-neoplastic lesions. Neoplastic lesions are caused by direct infiltration of the skin by the lymphoplasmacytic cells; non-neoplastic lesions are secondary to paraproteinemia [6,7]. The non-neoplastic lesions are more common and can be divided into the following three subtypes based on the aetiology: hyperviscosity syndrome (acral purpura, mucosal bleeding, peripheral oedema); associations with cryoglobulinemia (acrocyanosis, Raynaud phenomenon, cold hypersensitivity, livedo reticularis, leukocytoclastic vasculitis); and those secondary to paraproteinemia (immunoglobulin M bullous dermatosis, macroglobulinemia cutis, and erythematous papules associated with WM) [6-8]. Based on previous reports, cutaneous manifestations occur years after the initial WM diagnosis and may only affect 5% of WM patients [6]. In our case, the patient presented with a subcutaneous penile mass in the absence of cutaneous manifestations. It is most likely that the relapse of WM was detected early enough to prevent integumentary involvement. Nonetheless, the mass can be characterised as neoplastic from the presence of lymphoplasmacytic cells (Figure 3), making this a rare finding due to the particular composition and location of the mass.

Penile presentation of WM is extremely rare, with only one case identified by our literature search [3]. Additionally, in the case described by Oliveria et al. [3], the lesion was a cutaneous manifestation described as a 4 cm verrucous ulcer. Our patient had a history of WM with recurrence occurring at the base of the penis as a subcutaneous mass. We hypothesise that the involvement was most likely from a hematogenous or lymphatic route rather than an extension from an existing tumour. It is challenging to determine the exact mechanism since lymphadenopathy may not be present in some cases. Of note, it is also difficult to explain why the lesion presented at the base of the penis rather than a blood supply-rich structure. For any patient presenting with a penile lesion, it is imperative to obtain a thorough sexual and oncological history, and then perform a biopsy if suspicion of penile cancer remains high. A diagnosis of the most common penile cancer, squamous cell carcinoma, can only be made once keratin pearls are visualised on biopsy; the lack of keratin structures should suggest a diagnosis of metastasis, sarcoma, or lymphoma [9]. Immunohistochemical analysis can be used to differentiate lymphomas and sarcomas with imaging such as CT, MRI, and positron emission tomography be reserved for disease staging [10].

Even though non-squamous penile malignancy is rare, it is imperative to understand the treatment for these non-Hodgkin lymphomas, typically consisting of chemotherapy and immunotherapy, with surgery reserved as the last option. From our literature review, the management varies between initiating systemic therapy and delaying surgery as the last option or utilising surgery to remove most of the penile mass and starting systemic chemotherapy [9,11,12]. The overall consensus remains to initiate systemic chemotherapy with six CHOP (cyclophosphamide, hydroxydaunorubicin, oncovin, prednisone) cycles; some variations of CHOP include adding rituximab. Gertz reported on recent guidelines for WM treatment, whether it is primary or secondary in origin, with the utilisation of six cycles of CHOP and one of rituximab if the disease burden is low. In the presence of hyperviscosity symptoms, constitutional, and profound cytopenias, a regimen of four to six cycles of bendamustine and rituximab must be used [13]. In the case of a secondary lymphoma, just as in our case, systemic therapy must be initiated to avoid the risk of continued spread [9]. Our patient was treated with rituximab and ibrutinib and continues to be monitored for any new symptoms. Because of the rarity of this pathological entity, a high index of suspicion is required to diagnose extramedullary involvement of WM and to institute appropriate treatment to avoid potentially unnecessary mutilating surgery.

## Conclusions

WM with subcutaneous penile involvement is extremely rare, and there have been no other reported cases. This case provides a clinical example of why clinicians should consider lymphoma relapse in a patient who presents with a mass on the base of the penis and has a history of lymphoma. While much less common than squamous cell carcinoma of the penis, WM should be kept on the differential diagnosis.
